# Greater serum carotenoid levels associated with lower prevalence of nonalcoholic fatty liver disease in Chinese adults

**DOI:** 10.1038/srep12951

**Published:** 2015-08-10

**Authors:** Yi Cao, Cheng Wang, Jun Liu, Zhao-min Liu, Wen-hua Ling, Yu-ming Chen

**Affiliations:** 1 Guangdong Provincial Key Laboratory of Food, Nutrition and Health, School of Public Health, Sun Yat-sen University, Guangzhou, People’s Republic of China; 2 Department of Medical Records, The first Affiliated Hospital of Sun Yat-sen University, Guangzhou, People’s Republic of China; 3 Division of Family Medicine and Primary Care, Jockey Club School of Public Health and Primary Care, the Chinese University of Hong Kong, Hong Kong SAR

## Abstract

Previous studies have suggested that serum carotenoids may be inversely associated with liver injury, but limited data are available from population-based studies. We examined the relationship between serum carotenoid levels and the prevalence of nonalcoholic fatty liver disease (NAFLD) in Chinese adults. A total of 2935 participants aged 40–75 years were involved in this community-based cross-sectional study. General information, lifestyle factors, serum levels of carotenoid and the presence and degree of NAFLD were determined. After adjusting for potential covariates, we observed a dose-dependent inverse association between NAFLD risk and each individual serum carotenoid and total carotenoids (all p-values < 0.001). The ORs of NAFLD for the highest (vs. lowest) quartile were 0.44 (95% CI 0.35, 0.56) for α-carotene, 0.32 (95% CI 0.25, 0.41) for β-carotene, 0.62 (95% CI 0.49, 0.79) for β-cryptoxanthin, 0.54 (95% CI 0.42, 0.68) for lycopene, 0.56 (95% CI 0.44, 0.72) for lutein + zeaxanthin and 0.41 (95% CI 0.32, 0.53) for total carotenoids. Higher levels of α-carotene, β-carotene, lutein + zeaxanthin and total carotenoids were significantly associated with a decrease in the degree of NAFLD (p-trend: < 0.001 to 0.003). Serum carotenoids are inversely associated with prevalence of NAFLD in middle aged and elderly Chinese.

Non-alcoholic fatty liver disease (NAFLD) refers to a wide spectrum of conditions ranging from benign accumulation of fat in hepatocytes to non-alcoholic steatohepatitis, cirrhosis and end-stage liver disease. NAFLD is the most common liver disease and an alarming public health problem globally^1^. The main hypothesis describing the pathogenesis of NAFLD is a two-hit theory consisting of hepatic fat accumulation, followed by liver injury caused by oxidative stress^2^,^3^. As oxidative stress is thought to play a key role in the pathogenesis of NAFLD, antioxidants would be expected to reduce the risk of NAFLD.

Carotenoids are a group of diet-derived phytochemicals with antioxidant and anti-inflammatory properties that have attracted substantial interest because of their capacity to reduce the risks of relevant diseases^4^,^5^. Although appealing in theory, the evidence supporting a protective role for serum carotenoids against NAFLD in human is limited^6^,^7^,^8^,^9^. Most previous studies have investigated the association between carotenoids and NAFLD using liver aminotransferase, a poor biomarker for NAFLD^10^, and the results have been conflicting^6^,^7^,^8^,^9^. In the NHANES III, higher levels of serum carotenoids were associated with lower presence of apparent liver injury, as indicated by elevated alanine transaminase^6^. A Japanese study indicated that the inverse association of aminotransferase levels with β-cryptoxanthin and β-carotene was more pronounced in hyperglycemic than in normal participants^7^. No significant relationships between blood carotenoids and aminotransferase levels were noted in two cross-sectional studies, although serum carotenoid levels were significantly lower in NAFLD patients diagnosed by biopsy or magnetic resonance imaging compared with controls^8^,^9^. Liver tissue evaluation is the only unequivocal means of diagnosing NAFLD, but clearly cannot be used in large epidemiological studies or clinical screenings^2^. Ultrasound evaluation of NAFLD is an established tool with acceptable sensitivity and specificity for detecting fatty liver disease in population-based studies^11^. However, few studies have examined the associations between circulating carotenoids and NAFLD assessed by ultrasound, magnetic resonance imaging^9^ or tissue evaluation^8^. Therefore, the hypothesized associations between circulating carotenoids and NAFLD remain speculative.

The prevalence of NAFLD is known to vary according to ethnicity and increases substantially in parallel with regional trends in over-nutrition, obesity and type 2 diabetes mellitus^2^,^12^,^13^. A previous study found that the carotenoid-obesity association was weaker in Black than in White participants^4^, suggesting potential ethnic differences between carotenoid and NAFLD. More studies are needed to clarify the role of serum carotenoids on NAFLD in humans with different ethnic backgrounds and lifestyles. To address this, we tested the hypothesis that higher levels of serum carotenoids are associated with a lower risk of NAFLD using ultrasonography diagnosis in middle-aged and elderly Chinese people.

## Methods

### Study population

This community-based cross-sectional study was based on the first follow-up of a cohort study designed to investigate the environmental and genetic determinants of cardiometabolic endpoints and osteoporosis^14^. Between July 2008 and June 2010, 3216 participants aged 40–75 years were recruited by posting local advertisements, through health talks, or from referrals in the local community in Guangzhou, China. Participants who reported cancer, Alzheimer’s disease, cardiovascular disease or renal failure were excluded before the start of the study, leaving 3169 participants. Of these, 2465 were followed up between April 2011 and January 2013. During this period, an additional 871 participants were recruited in the same way as described above. We excluded those with missing data for serum carotenoid (n = 264) or abdominal ultrasonography (n = 108). We also excluded participants with excessive alcohol intake (more than 140 g weekly for men and 70 g weekly for women, n = 12) or self-reported viral hepatitis (n = 17). Finally, 2935 participants, comprising 2215 followed up from the original cohort and 720 newly recruited participants, were included in this analysis.

The Ethics Committee of the School of Public Health at Sun Yat-sen University approved the study protocol. Written informed consent was obtained from all of the participants at initial enrollment and at follow-up. The study was therefore performed in accordance with the ethical standards laid down in the 1964 Declaration of Helsinki and its later amendments.

### Data collection

Participants’ socio-demographic characteristics (e.g., age, sex, household income), medications, health-related lifestyle factors (e.g., smoking, tea drinking and physical activity) and history of chronic disease were collected by trained staff using a structured questionnaire by means of face-to-face interviews. Weight and height were measured while participants wore lightweight clothes and no shoes. Body mass index (BMI) was calculated as weight (kg)/height (m^2^). A 24-h physical activity questionnaire was used to estimate daily physical activity, and the metabolic equivalent (MET) intensity was calculated^15^. Participants’ usual dietary intakes were assessed using a validated quantitative food-frequency questionnaire including 79 items^16^. Energy intake and other nutrients were estimated based on the 2004 Chinese Food Composition Table^17^.

### Laboratory assay

A venous blood sample was obtained after overnight fasting. The serum was separated into several aliquots and stored at −80 °C within 2 hours. Serum glucose was measured using colorimetric methods in a Hitachi 7600-010 automated analyzer (Hitachi, Tokyo, Japan). Impaired fasting glucose in our study was defined as serum levels of glucose ≥ 6.1 mmol/L or diagnosed as diabetes before. Serum concentrations of α-carotene, β-carotene, β-cryptoxanthin, lycopene and lutein/zeaxanthin were simultaneously analyzed using reversed-phase high-performance liquid chromatography^18^, using α-tocopherol acetate as the internal standard. The carotenoids were extracted twice using hexane-BHT solution. The organic layer was removed, evaporated to dryness under nitrogen, dissolved in 200 μl of mobile phase B and transferred to a microvial for automatic injection. The chromatography system was fitted with a C18 analytical column (SHISEIDO, Japan) and a Waters 2998 diode-array detector (Waters, MA, USA). The mobile phase comprised A (acetonitrile-methanol-tetrahydrofuran-ammonium acetate 85:5:5:5, v/v) and B (acetonitrile-methanol-tetrahydrofuran-ammonium acetate 55:35:5:5, v/v). The combined concentration of lutein and zeaxanthin was used in the analyses because these methods do not discriminate lutein from zeaxanthin. A pooled plasma sample was analyzed with each batch of samples, with a day-to-day coefficient of variation of 7.8% for α-carotene, 8.6% for β-carotene, 9.7% for β-cryptoxanthin, 10.6% for lycopene and 8.0% for lutein + zeaxanthin.

### Abdominal ultrasonography

The diagnosis of NAFLD was based on an abdominal ultrasound using a Doppler sonography machine (Sonoscape SSI-5500, Shenzhen, China) with a 3.5 MHz probe. All ultrasound examinations were performed by a single experienced radiologist who was blinded to the participant’s details, including laboratory and clinical data, at the time of the procedure.

NAFLD was diagnosed based on standard criteria issued by the Fatty Liver Disease Study Group of the Chinese Liver Disease Association, and the degree of steatosis was assessed semi-quantitatively (rated as absent, mild, moderate or severe) on the basis on hepatorenal echo contrast, liver brightness, deep attenuation and vascular blurring^19^.

### Statistical analysis

The data are presented here as means ± SDs for continuous variables and frequencies (percentages) for categorical variables. *t-*tests and Chi-square tests were used to analyze the differences in the data stratified by sex or the presence of NAFLD. All dietary nutrient intake data were adjusted for energy intake using a residual method. We used logistic regression analyses to estimate the ORs and 95% confidence intervals (CIs) for the risk of NAFLD with increasing quartiles of serum carotenoids, using the lowest quartile as the reference group. We adjusted for age, sex and energy intake in model 1. To investigate the independent association, we further adjusted for BMI, physical activity, household income, multivitamin use, smoking, tea drinking, dietary intake of cholesterol and fiber, saturated fatty acid to polyunsaturated fatty acid ratio, and serum glucose levels. The relationships between serum carotenoids and the degree of NAFLD (absent, mild, moderate or severe) were also investigated using analysis of covariance, and the variables were adjusted as in Model 2.

Analyses stratified by sex, BMI, hyperglycemia, smoking and income were conducted to examine whether the above associations would be modified by these factors. A two-tailed p-value < 0.05 was considered statistically significant. Since multiple testing could amplify type I errors, the significance levels were adjusted by Bonferroni’s correction to counteract the effect of multiple tests (α/n). All of the statistical procedures were performed using SPSS Statistics (version 20.0, SPSS Inc, Chicago, IL).

## Results

The prevalence of NAFLD in this study, as diagnosed with ultrasound, was estimated to be 50.6%. The characteristics of the participants are presented in Table 1. The study involved 2935 participants, of whom 2006 were women with a mean age of 59.9 y and 929 were men with a mean age of 62.4 y. Compared with the men, the women in the cohort had higher levels of serum carotenoids, but lower BMIs and fasting serum glucose (all p < 0.001). Participants with NAFLD were more likely to have high BMIs and fasting serum glucose levels, and showed lower physical activity and lower serum carotenoid concentration (all p < 0.001).

**Figure 1:**
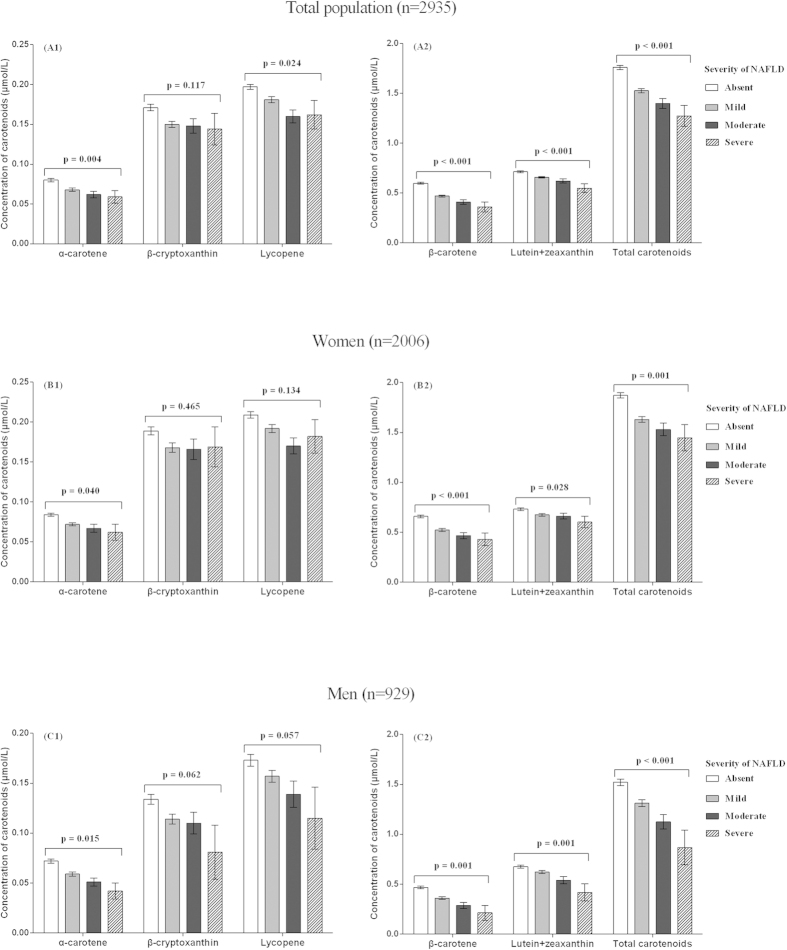
Multivariable adjusted means (SEM) of serum carotenoids (μmol/l) according to the severity of nonalcoholic fatty liver disease (NAFLD). Means (SEM) were adjusted for age, energy intake, body mass index, physical activity, household income, multivitamin user, smoking, tea drinker, serum levels of glucose, dietary intake of carbohydrate, cholesterol, fiber, saturated fatty acid to polyunsaturated fatty acid ratio using analysis of covariance. *p*-values here stand for linear trend between serum levels of carotenoids and severity of NAFLD.

**Figure 2:**
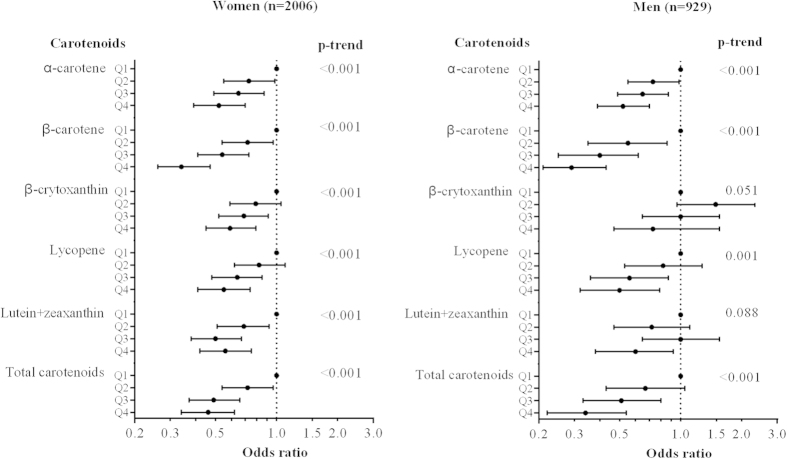
Adjusted odds ratios (95%CIs) for nonalcoholic fatty liver disease according to quartiles (Q1-Q4) of serum carotenoids. Odds ratios (95% CIs) were adjusted for age, energy intake, body mass index, physical activity, household income, multivitamin user, smoking, tea drinker, serum levels of glucose, dietary intake of carbohydrate, cholesterol, fiber, saturated fatty acid to polyunsaturated fatty acid ratio.

As shown in Table 2, after adjusting for age, sex and energy intake, serum carotenoid levels were inversely associated with the prevalence of NAFLD (all p < 0.001). Similar associations were observed after further adjustment of variables in model 2. The ORs of NAFLD for the highest (vs. lowest) quartile were 0.44 (95% CI 0.35, 0.56) for α-carotene, 0.32 (95% CI 0.25, 0.41) for β-carotene, 0.62 (95% CI 0.49, 0.79) for β-cryptoxanthin, 0.54 (95% CI 0.42, 0.68) for lycopene, 0.56 (95% CI 0.44, 0.72) for lutein and zeaxanthin in combination and 0.41 (95% CI 0.32, 0.53) for total carotenoids (all p-values < 0.001). As shown in Fig. 1, the mean serum levels of α-carotene, β-carotene, lutein + zeaxanthin and total carotenoids decreased significantly with the presence and increased severity of NAFLD after adjustments for the variables mentioned above in model 2 (p-value range: < 0.001–0.004, α = 0.05/6 tests). We observed consistent results in both women and men (Figs 1 and 2), and for each of the five individual carotenoids (Table 2, Fig. 1).

No significant interactions were found between individual carotenoids or total carotenoids and any of the stratified variables, including sex, BMI, impaired fasting glucose, smoking and household income, in relation to NAFLD (all p for interaction > 0.002, α = 0.05/30 tests; see Supplemental Table S1).

## Discussion

We found an inverse association between serum levels of carotenoids and the prevalence of NAFLD and determined a dose-response in a large, community-based middle-aged and elderly Chinese population. Moreover, serum levels of α-carotene, β-carotene, lutein + zeaxanthin and total serum carotenoids were found to decrease significantly with an increase in the degree of NAFLD. Our findings provide evidence for a favorable association between serum carotenoids and the prevention of NAFLD.

One of the potential pathophysiological mechanisms linking serum carotenoid to NAFLD is the counterbalancing of oxidative stress. In the pathogenesis of NAFLD, the increasing accumulation of fatty acids in hepatocyte could ultimately result in reactive oxygen species being produced in mitochondria, peroxisomes and the cytochrome P450, CYP2E1 and CYP4A systems, causing lipid peroxidation and cytokine release leading to hepatocyte injury^3^,^20^. Carotenoids are known for their antioxidative activities, including in quenching free radicals, reducing damage from reactive oxidant species and inhibiting lipid peroxidation^21^. Carotenoids may also mediate their protective effects against NAFLD through other mechanisms, such as enhancing gap junction communication, reducing inflammation or modulating gene expression^21^,^22^,^23^.

We are unaware of any other study that has reported a dose-dependent inversely association between serum carotenoids and the prevalence of NAFLD diagnosed by imaging in a large population. Clinical evidence for the protective properties of such antioxidants against NAFLD has largely been limited to vitamin E intervention studies, and the results have been inconclusive^24^,^25^. A robust histological improvement was observed in participants who received vitamin E (800 IU per day) for 96 weeks in a study including 247 adults with nonalcoholic steatohepatitis (NASH)^25^, but another clinical trial containing 173 children and adolescents failed to reproduce this finding^24^. To the best of our knowledge, no interventional study has reported the effects of carotenoids on the risk or progress of NAFLD as the primary or secondary endpoints. A cross-sectional study of 57 biopsy-proven NAFLD patients revealed that their serum carotenoid levels were significantly decreased compared to healthy controls^8^. Similar results were found in another cross-sectional study^9^. NAFLD is considered to be the hepatic manifestation of metabolic syndrome, with insulin resistance being the prevailing pathogenetic mechanism^26^. The NHANES III study, including 8808 adults, reported that serum carotenoids were significantly decreased in participants with metabolic syndrome compared with those without^27^. Similar results have since been found in adolescents^28^ and confirmed in other cross-sectional studies^29^,^30^. In our study, serum carotenoid levels were not only inversely associated with the presence of NAFLD, but also decreased significantly with increased NAFLD severity. Further prospective studies are needed to confirm these findings.

We did not measure the serum alanine transaminase value that has long been used as a marker of liver injury^31^. Previous studies have suggested that NAFLD and even NASH with fibrosis and/or cirrhosis may appear histologically in the normal aminotransferase status range^32^. In addition, it has recently been shown that liver apoptosis and oxidative stress, the main processes contributing to the progression of NAFLD, have no relationship with alanine transaminase levels^10^. The ultrasonography used in this study is an established tool for NAFLD screening with acceptable sensitivity (84%) and specificity (95%) compared with liver biopsy^33^, and is non-invasive, inexpensive and widely available^11^. The prevalence of NAFLD among middle-aged and elderly Chinese people was 50.6% in this study. Compared with Caucasians, Asians have increased amounts of visceral fat, which secretes more tumor necrosis factor and interleukin-6 and less adiponectin^34^. As a result, NAFLD risks can be equivalent despite lower BMI^35^.

Our study possesses several advantages. The large community-based study sample provided us with sufficient power to detect relatively small effects. We used abdominal ultrasonography, rather than aminotransferase, for the classification of NAFLD. We investigated the relationship of five individual carotenoids and the total carotenoid level with the presence of NAFLD. The consistent relationships found provided strong evidence for the inverse associations proposed. The measurement of serum carotenoids allowed us to objectively and accurately assess internal carotenoid exposures and avoid the variations in bioavailability among individuals based on diet. Finally, we carefully adjusted for various covariates to avoid potential confounding effects.

Some limitations merit consideration. First, we were not able to infer a causal relationship between serum carotenoids and risk of NAFLD due to the cross-sectional study design. However, the observed favorable association was unlikely to be an inverse causal relationship since prevalent NAFLD patients tended to have healthy lifestyles following doctor suggestions. Second, high serum carotenoid levels reflect high intakes of fruits and vegetables, and could be a surrogate for healthy nutrition (e.g., intake of other antioxidants) and other lifestyle factors. We cannot completely rule out the effect of these factors, although we carefully adjusted for a variety of relevant covariates. Third, the non-random study sample may limit the generalizability of our findings. However, the associations found were not affected significantly by sex, BMI, hyperglycemia, smoking or household income, suggesting that they are fairly generalizable with respect to these factors. Finally, the existence of oxidative stress depends on the relative balance of reactive oxygen species and all antioxidant defenses within the microenvironment. We did not determine biomarkers for oxidative stress or circulating antioxidative levels.

In conclusion, we found that serum carotenoid levels were inversely associated with the risk of the presence and the degree of NAFLD in middle-aged and elderly Chinese people. Our findings add to the limited data available on the favorable associations between carotenoids and reduced NAFLD risk. Special attention should be given to carotenoids in public health interventions for the prevention of NAFLD.

## Additional Information

**How to cite this article**: Cao, Y. *et al.* Greater serum carotenoid levels associated with lower prevalence of nonalcoholic fatty liver disease in Chinese adults. *Sci. Rep.* **5**, 12951; doi: 10.1038/srep12951 (2015).

## Supplementary Material

Supplementary Information

## Figures and Tables

**Table 1:** Characteristic of participants in Guangzhou, China.

	Women	Men	p-value	Non-NAFLD	NAFLD	p-value
N, (%)	2006 (68.3)	929 (31.7)		1449 (49.4)	1486 (50.6)	
age, y	59.9 ± 5.5	62.4 ± 6.4	< 0.001	60.6 ± 6.1	60.8 ± 5.7	0.317
BMI, kg/m^2^	23.4 ± 5.4	23.9 ± 3.0	< 0.001	22.1 ± 2.6	25.0 ± 3.0	< 0.001
Smoking, N (%)^a^	7.0 (0.3)	325 (35.0)	< 0.001	158 (10.9)	174 (11.7)	0.491
Tea drinker, N (%)^b^	963 (48.1)	690 (74.3)	< 0.001	757 (52.3)	896 (60.3)	< 0.001
Multivitamin user, N (%)^c^	441 (22.0)	120 (12.9)	< 0.001	276 (19.1)	285 (19.2)	0.921
Household income, yuan/month/person, N (%)			0.463			0.060
< 4000	1118 (55.7)	496 (53.4)		829 (57.2)	795 (53.5)	
4000–6000	449 (22.4)	216 (23.3)		322 (22.2)	334 (22.5)	
> 6000	439 (21.9)	217 (23.4)		298 (20.6)	357 (24.0)	
PA, MET-h/day^d^	34.1 ± 5.4	33.8 ± 6.1	0.213	34.3 ± 5.7	33.6 ± 1.2	< 0.001
FBG, mmol/l	4.96 ± 1.12	5.11 ± 1.14	0.001	4.89 ± 1.00	5.13 ± 1.23	< 0.001
Dietary intake, /day (mean ± SD)						
Energy, kcal	1512 ± 423	1773 ± 497	< 0.001	1583 ± 445	1607 ± 481	0.162
Carbohydrate, g	213 ± 61	258 ± 77	< 0.001	226 ± 68	229 ± 72	0.281
Cholesterol, mg	348 ± 180	366 ± 167	0.017	355 ± 164	355 ± 187	0.735
Fiber, g	11.5 ± 4.85	11.4 ± 4.76	0.865	11.4 ± 4.6	11.6 ± 5.0	0.452
SFA, g	12.5 ± 5.3	13.9 ± 6.3	< 0.001	12.8 ± 5.5	13.1 ± 5.9	0.202
PUFA, g	12.4 ± 5.9	13.4 ± 6.1	< 0.001	12.5 ± 5.5	12.9 ± 6.0	0.042
Serum antioxidants, μmol/l (mean ± SD)						
Lutein/zeaxanthin	0.701 ± 0.349	0.637 ± 0.317	< 0.001	0.732 ± 0.350	0.631 ± 0.324	< 0.001
β-cryptoxanthin	0.178 ± 0.155	0.122 ± 0.101	< 0.001	0.173 ± 0.147	0.148 ± 0.137	< 0.001
Lycopene	0.199 ± 0.131	0.162 ± 0.118	< 0.001	0.201 ± 0.131	0.173 ± 0.124	< 0.001
α-carotene	0.078 ± 0.059	0.064 ± 0.048	< 0.001	0.082 ± 0.061	0.064 ± 0.049	< 0.001
β-carotene	0.586 ± 0.388	0.402 ± 0.298	< 0.001	0.623 ± 0.404	0.434 ± 0.311	< 0.001
Total carotenoids	1.74 ± 0.82	1.39 ± 0.66	< 0.001	1.81 ± 0.82	1.45 ± 0.71	< 0.001

Abbreviation: BMI, body mass index; PA, physical activity; FBG, fasting blood glucose; SFA, saturated fatty acid; PUFA, polyunsaturated fatty acid.

^a^Smoking: ≥ 1 cigarette/d in the past year.

^b^Tea drinkers: ≥ 1 cup/week in the past year.

^c^Multivitamin user: ≥ 30 times in the past year.

^d^PA: Physical activities, in metabolic equivalent (MET) hours per day.

**Table 2:** Odds ratios (95%CIs) for non-alcoholic fatty liver disease according to quartiles of serum carotenoid in total participants.

	Quartiles by serum carotenoids				
	Q1	Q2	Q3	Q4	p-trend
α-carotene					
Median, μmol/l	0.026	0.047	0.074	0.146	
Case, n (%)	459 (62.5)	399 (54.4)	352 (47.9)	276 (37.7)	
OR 1	1.00	0.71 (0.58,0.88)	0.55 (0.45,0.68)	0.36 (0.29,0.44)	< 0.001
OR 2	1.00	0.69 (0.56,0.88)	0.59 (0.46,0.75)	0.44 (0.35,0.56)	< 0.001
β-carotene					
Median, μmol/l	0.174	0.352	0.556	1.028	
Case, n (%)	500 (68.2)	406 (55.3)	342 (46.5)	238 (32.5)	
OR 1	1.00	0.58 (0.47,0.72)	0.41 (0.33,0.50)	0.22 (0.18,0.28)	< 0.001
OR 2	1.00	0.65 (0.51,0.83)	0.48 (0.38,0.62)	0.32 (0.25,0.41)	< 0.001
β-cryptoxanthin					
Median, μmol/l	0.052	0.091	0.154	0.345	
Case, n (%)	419 (57.2)	399 (54.4)	351 (47.8)	317 (43.2)	
OR 1	1.00	0.90 (0.73,1.11)	0.69 (0.56,0.85)	0.57 (0.46,0.70)	< 0.001
OR 2	1.00	0.96 (0.76,1.22)	0.76 (0.60,0.96)	0.62 (0.49,0.79)	< 0.001
Lycopene					
Median, μmol/l	0.074	0.13	0.191	0.352	
Case, n (%)	448 (61.1)	396 (54.0)	330 (44.9)	312 (42.6)	
OR 1	1.00	0.75 (0.61,0.92)	0.52 (0.42,0.64)	0.47 (0.38,0.58)	< 0.001
OR 2	1.00	0.81 (0.64,1.03)	0.61 (0.48,0.77)	0.54 (0.42,0.68)	< 0.001
Lutein + zeaxanthin					
Median, μmol/l	0.321	0.529	0.731	1.14	
Case, n (%)	457 (62.3)	378 (51.5)	347 (47.2)	304 (41.5)	
OR 1	1.00	0.64 (0.52,0.79)	0.54 (0.44,0.67)	0.43 (0.35,0.53)	< 0.001
OR 2	1.00	0.69 (0.55,0.88)	0.63 (0.49,0.79)	0.56 (0.44,0.72)	< 0.001
Total carotenoids					
Median, μmol/l	0.78	1.29	1.77	2.68	
Case, n (%)	481 (65.6)	404 (55.0)	331 (45.0)	270 (36.8)	
OR 1	1.00	0.64 (0.52,0.80)	0.43 (0.35,0.53)	0.31 (0.25,0.38)	< 0.001
OR 2	1.00	0.69 (0.54,0.88)	0.50 (0.39,0.63)	0.41 (0.32,0.53)	< 0.001

OR 1: odds ratio (95%CI), adjusted for age, sex and energy intake. OR 2: odds ratio (95%CI), adjusted for variables in OR 1 plus body mass index, physical activity, household income, multivitamin user, smoking, tea drinker, serum levels of glucose, dietary intake of carbohydrate, cholesterol, fiber, saturated fatty acid to polyunsaturated fatty acid ratio.
