# Supplementary material for: Greater serum carotenoid levels associated with lower prevalence of nonalcoholic fatty liver disease in Chinese adults
Source: Sci Rep. 2015 Aug 10;5:12951. doi: 10.1038/srep12951 (PMC4530335; doi:10.1038/srep12951)
Supplement: Supplementary Information [file srep12951-s1.doc]

**Title:** Greater serum carotenoid levels associated with lower prevalence of nonalcoholic fatty liver disease in Chinese adults

**Authors:** Yi Cao, Cheng Wang, Jun Liu, Zhao-min Liu, Wen-hua Ling, Yu-ming Chen

**Supplemental Table S1.** **Multivariable adjusted ORs and 95%CIs of NAFLD according to quartiles of serum carotenoid stratified by sex, BMI, hyperglycemia, smoking and household income ^a^**

|  | Q1 | | | Q2 | | | | Q3 | Q4 | p - |
| --- | --- | --- | --- | --- | --- | --- | --- | --- | --- | --- |
|  |  | | |  | | | |  |  | interaction |
| ***α-carotene, μmol/l*** |  | | |  | | | |  |  |  |
| Sex |  | | |  | | | |  |  | 0.114 |
| women | 1.00 | | | 0.73(0.55,0.98) | | | | 0.65(0.49,0.87) | 0.52(0.39,0.70) |  |
| men | 1.00 | | | 0.58(0.37,0.91) | | | | 0.44(0.28,0.69) | 0.30(0.19,0.48) |  |
| Body mass index |  | | |  | | | |  |  | 0.154 |
| < 24.0 | 1.00 | | | 0.62(0.45,0.85) | | | | 0.64(0.47,0.88) | 0.46(0.34,0.63) |  |
| ≥24.0 | 1.00 | | | 0.80(0.54,1.17) | | | | 0.51(0.35,0.74) | 0.39(0.26,0.74) |  |
| Impaired fasting glucose | | |  |  | | | |  |  | 0.193 |
| <6.1 | 1.00 | | | 0.71(0.55,0.91) | | | | 0.57(0.44,0.73) | 0.46(0.36,0.60) |  |
| ≥6.1 | 1.00 | | | 0.67(0.31,1.41) | | | | 0.84(0.40,1.77) | 0.30(0.14,0.66) |  |
| Smoking |  | | |  | | | |  |  | 0.593 |
| no | 1.00 | | | 0.70(0.54,0.91) | | | | 0.62(0.48,0.80) | 0.45(0.35,0.59) |  |
| yes | 1.00 | | | 0.65(0.33,1.28) | | | | 0.43(0.21,0.87) | 0.40(0.19,0.83) |  |
| Household income, yuan/month/person | | | | | | |  |  |  | 0.908 |
| < 4000 | 1.00 | | | 0.70(0.51,0.96) | | | | 0.58(0.42,0.80) | 0.44(0.31,0.61) |  |
| 4000-6000 | 1.00 | | | 0.61(0.36,1.05) | | | | 0.60(0.35,1.02) | 0.32(0.18,0.56) |  |
| > 6000 | 1.00 | | | 0.72(0.42,1.22) | | | | 0.58(0.35,0.95) | 0.55(0.33,0.91) |  |
| ***β-carotene, μmol/l*** |  | | |  | | | |  |  |  |
| Sex |  | | |  | | | |  |  | 0.589 |
| women | 1.00 | | | 0.72(0.54,0.96) | | | | 0.54(0.41,0.73) | 0.34(0.26,0.47) |  |
| men | 1.00 | | | 0.55(0.35,0.86) | | | | 0.40(0.25,0.62) | 0.29(0.18,0.46) |  |
| Body mass index |  | | |  | | | |  |  | 0.023 |
| < 24.0 | 1.00 | | | 0.61(0.44,0.84) | | | | 0.57(0.42,0.78) | 0.38(0.28,0.53) |  |
| ≥24.0 | 1.00 | | | 0.75(0.51,1.11) | | | | 0.40(0.27,0.58) | 0.24(0.16,0.36) |  |
| Impaired fasting glucose | | |  |  | | | |  |  | 0.545 |
| <6.1 | 1.00 | | | 0.67(0.52,0.87) | | | | 0.47(0.36,0.61) | 0.32(0.24,0.41) |  |
| ≥6.1 | 1.00 | | | 0.45(0.21,0.94) | | | | 0.59(0.29,1.22) | 0.37(0.17,0.80) |  |
| Smoking |  | | |  | | | |  |  | 0.178 |
| no | 1.00 | | | 0.67(0.52,0.88) | | | | 0.51(0.40,0.66) | 0.32(0.24,0.42) |  |
| yes | 1.00 | | | 0.57(0.29,1.13) | | | | 0.31(0.15,0.65) | 0.39(0.18,0.83) |  |
| Household income, yuan/month/person | | | | | | |  |  |  | 0.061 |
| < 4000 | 1.00 | | | 0.63(0.46,0.87) | | | | 0.53(0.38,0.74) | 0.31(0.22,0.44) |  |
| 4000-6000 | 1.00 | | | 0.33(0.18,0.58) | | | | 0.26(0.15,0.47) | 0.18(0.10,0.32) |  |
| > 6000 | 1.00 | | | 1.23(0.72,2.10) | | | | 0.61(0.37,1.01) | 0.52(0.31,0.89) |  |
| ***β-cryptoxanthin, μmol/l*** | |  | |  | | | |  |  |  |
| Sex |  | | |  | | | |  |  | 0.145 |
| women | 1.00 | | | 0.79(0.59,1.05) | | | | 0.69(0.52,0.91) | 0.59(0.45,0.79) |  |
| men | 1.00 | | | 1.49(0.96,2.32) | | | | 1.00(0.65,1.55) | 0.73(0.47,1.55) |  |
| Body mass index |  | | |  | | | |  |  | 0.086 |
| < 24.0 | 1.00 | | | 0.77(0.47,1.05) | | | | 0.68(0.50,0.92) | 0.60(0.44,0.82) |  |
| ≥24.0 | 1.00 | | | 1.33(0.90,1.97) | | | | 0.87(0.59,1.26) | 0.65(0.45,0.93) |  |
| Impaired fasting glucose | | |  |  | | | |  |  | 0.492 |
| <6.1 | 1.00 | | | 0.94(0.73,1.21) | | | | 0.71(0.55,0.92) | 0.62(0.48,0.79) |  |
| ≥6.1 | 1.00 | | | 1.10(0.54,2.25) | | | | 1.46(0.68,3.14) | 0.61(0.29,1.31) |  |
| Smoking |  | | |  | | | |  |  | 0.793 |
| no | 1.00 | | | 0.93(0.72,1.20) | | | | 0.74(0.57,0.96) | 0.62(0.48,0.80) |  |
| yes | 1.00 | | | 1.09(0.56,2.14) | | | | 0.84(0.41,1.71) | 0.58(0.28,1.21) |  |
| Household income, yuan/month/person | | | | | | |  |  |  | 0.319 |
| < 4000 | 1.00 | | | 0.97(0.71,1.33) | | | | 0.78(0.57,1.07) | 0.63(0.46,0.87) |  |
| 4000-6000 | 1.00 | | | 0.82(0.48,1.41) | | | | 0.65(0.39,1.09) | 0.38(0.22,0.64) |  |
| > 6000 | 1.00 | | | 1.10(0.64.1.90) | | | | 0.83(0.49,1.43) | 0.99(0.58,1.70) |  |
| ***Lycopene, μmol/l*** |  | | |  | | | |  |  |  |
| Sex |  | | |  | | | |  |  | 0.856 |
| women | 1.00 | | | 0.82(0.62,1.10) | | | | 0.64(0.48,0.85) | 0.55(0.41,0.74) |  |
| men | 1.00 | | | 0.82(0.53,1.28) | | | | 0.56(0.36,0.87) | 0.50(0.32,0.79) |  |
| Body mass index |  | | |  | | | |  |  | 0.428 |
| < 24.0 | 1.00 | | | 0.95(0.70,1.30) | | | | 0.67(0.49,0.92) | 0.63(0.46,0.86) |  |
| ≥24.0 | 1.00 | | | 0.64(0.43,0.94) | | | | 0.54(0.37,0.80) | 0.45(0.30,0.66) |  |
| Impaired fasting glucose | | |  |  | | | |  |  | 0.193 |
| <6.1 | 1.00 | | | 0.85(0.66,1.10) | | | | 0.64(0.50,0.83) | 0.53(0.41.0.68) |  |
| ≥6.1 | 1.00 | | | 0.70(0.34,1.44) | | | | 0.43(0.20,0.90) | 0.67(0.31,1.43) |  |
| Smoking |  | | |  | | | |  |  | 0.736 |
| no | 1.00 | | | 0.81(0.63,1.05) | | | | 0.59(0.46,0.76) | 0.55(0.42,0.71) |  |
| yes | 1.00 | | | 0.92(0.47,1.80) | | | | 0.88(0.44,1.78) | 0.46(0.22,0.96) |  |
| Household income, yuan/month/person | | | | | |  | |  |  | 0.244 |
| < 4000 | 1.00 | | | 0.89(0.65,1.21) | | | | 0.57(0.42,0.78) | 0.67(0.48,0.93) |  |
| 4000-6000 | 1.00 | | | 0.57(0.33,0.96) | | | | 0.50(0.29,0.86) | 0.30(0.18,0.53) |  |
| > 6000 | 1.00 | | | 0.88(0.50,1.56) | | | | 0.80(0.46,1.39) | 0.53(0.31,1.39) |  |
| ***Lutein+zeaxanthin, μmol/l*** | | | |  | | | |  |  |  |
| Sex |  | | |  | | | |  |  | 0.034 |
| women | 1.00 | | | 0.69(0.51,0.92) | | | | 0.50 (0.38,0.67) | 0.56(0.42,0.75) |  |
| men | 1.00 | | | 0.72(0.47,1.11) | | | | 1.00(0.65,1.55) | 0.60(0.38,0.92) |  |
| Body mass index |  | | |  | | | |  |  | 0.543 |
| < 24.0 | 1.00 | | | 0.65(0.47,0.89) | | | | 0.59(0.43,0.80) | 0.63(0.46,0.85) |  |
| ≥24.0 | 1.00 | | | 0.74(0.51,1.08) | | | | 0.68(0.46,0.99) | 0.46(0.31,0.69) |  |
| Impaired fasting glucose | | |  |  | | | |  |  | 0.970 |
| <6.1 | 1.00 | | | 0.70(0.54,0.90) | | | | 0.64(0.50,0.83) | 0.56(0.430.73) |  |
| ≥6.1 | 1.00 | | | 0.64(0.30,1.35) | | | | 0.53(0.26,1.08) | 0.53(0.25,1.11) |  |
| Smoking |  | | |  | | | |  |  | 0.153 |
| no | 1.00 | | | 0.67(0.52,0.86) | | | | 0.57(0.44,0.73) | 0.54(0.42,0.70) |  |
| yes | 1.00 | | | 1.02(0.50,2.07) | | | | 1.38(0.68,2.79) | 0.76(0.36,1.60) |  |
| Household income, yuan/month/person | | | | |  | | |  |  | 0.985 |
| < 4000 | 1.00 | | | 0.77(0.56,1.06) | | | | 0.69(0.50,0.95) | 0.60(0.43,0.82) |  |
| 4000-6000 | 1.00 | | | 0.60(0.36,1.02) | | | | 0.51(0.30,0.86) | 0.46(0.27,0.79) |  |
| > 6000 | 1.00 | | | 0.61(0.37,1.02) | | | | 0.53(0.32,0.89) | 0.54(0.32,0.92) |  |
| ***Total carotenoids, μmol/l*** | | | |  | | | |  |  |  |
| Sex |  | | |  | | | |  |  | 0.427 |
| women | 1.00 | | | 0.72(0.54,0.96) | | | | 0.49(0.37,0.66) | 0.46(0.34,0.62) |  |
| men | 1.00 | | | 0.67(0.43,1.05) | | | | 0.51(0.33,0.80) | 0.34(0.22,0.54) |  |
| Body mass index |  | | |  | | | |  |  | 0.207 |
| < 24.0 | 1.00 | | | 0.68(0.50,0.94) | | | | 0.52(0.38,0.72) | 0.49(0.36,0.67) |  |
| ≥24.0 | 1.00 | | | 0.73(0.49,1.07) | | | | 0.46(0.31,0.67) | 0.31(0.21,0.47) |  |
| Impaired fasting glucose | | |  |  | | | |  |  | 0.879 |
| <6.1 | 1.00 | | | 0.70(0.54,0.91) | | | | 0.49(0.38,0.63) | 0.41(0.32,0.53) |  |
| ≥6.1 | 1.00 | | | 0.59(0.24,1.24) | | | | 0.51(0.25,1.06) | 0.44(0.20,0.98) |  |
| Smoking |  | | |  | | | |  |  | 0.310 |
| no | 1.00 | | | 0.66(0.51,0.86) | | | | 0.50(0.39,0.65) | 0.40(0.31,0.52) |  |
| yes | 1.00 | | | 0.98(0.50,1.94) | | | | 0.47(0.23,0.95) | 0.52(0.24,1.10) |  |
| Household income, yuan/month/person | | | | |  | | |  |  | 0.412 |
| < 4000 | 1.00 | | | 0.77(0.56,1.06) | | | | 0.59(0.43,0.82) | 0.45(0.33,0.63) |  |
| 4000-6000 | 1.00 | | | 0.39(0.15,0.44) | | | | 0.26(0.15,0.44) | 0.22(0.13,0.39) |  |
| > 6000 | 1.00 | | | 0.80(0.47,1.36) | | | | 0.53(0.31,0.89) | 0.52(0.30,0.89) |  |

^a^ Adjusted for age, sex, energy intake, BMI, physical activity, Household income, multivitamin user, smoking, tea drinker, serum levels of glucose, dietary intake of carbohydrate, cholesterol, fiber, saturated fatty acid to polyunsaturated fatty acid ratio
